# Supplementary material for: Liver Transplantation in Patients with Portal Vein Thrombosis: Revisiting Outcomes According to Surgical Techniques
Source: J Clin Med. 2023 Mar 23;12(7):2457. doi: 10.3390/jcm12072457 (PMC10095520; doi:10.3390/jcm12072457)
Supplement: Supplementary file 1 [file jcm-12-02457-s001.zip › Supplementary Table S1.pdf]

Supplementary Table S1. Comparison of the main post-operative outcomes between the two groups of portal reconstruction (TTA versus Bypass)

	Overall (165)	TTA (123)	Bypass (42)	p-value
90 – day mortality; n(%)	21 (12.7)	12 (9.8)	9 (21.4)	0.050
1 – year mortality; n(%)	28 (17.0)	17 (13.8)	11 (26.2)	0.065
Overall mortality; n(%)	48 (29.1)	36 (29.3)	12 (28.6)	0.93
90 - day graft loss; n(%)	23 (13.9)	14 (11.4)	9 (21.4)	0.10
1 year graft loss; n(%)	30 (18.2)	19 (15.4)	11 (26.2)	0.12
Overall graft loss; n(%)	49 (29.7)	37 (30.1)	12 (28.6)	0.85
Post-LT portal thrombosis; n(%)	16 (9.7)	14 (11.4)	2 (4.8)	0.36
PNF; n(%)	6 (3.6)	3 (2.4)	3 (7.1)	0.17
Severe post-LT complications; n(%)	87 (52.7)	63 (51.2)	24 (57.1)	0.51
Retransplantation; n(%)	8 (4.8)	6 (4.9)	2 (4.8)	1

LT: liver transplantation; PNF: primary graft non function
