# Supplementary figures and images for: Liver Transplantation in Patients with Portal Vein Thrombosis: Revisiting Outcomes According to Surgical Techniques
Source: J Clin Med. 2023 Mar 23;12(7):2457. doi: 10.3390/jcm12072457 (PMC10095520; doi:10.3390/jcm12072457)

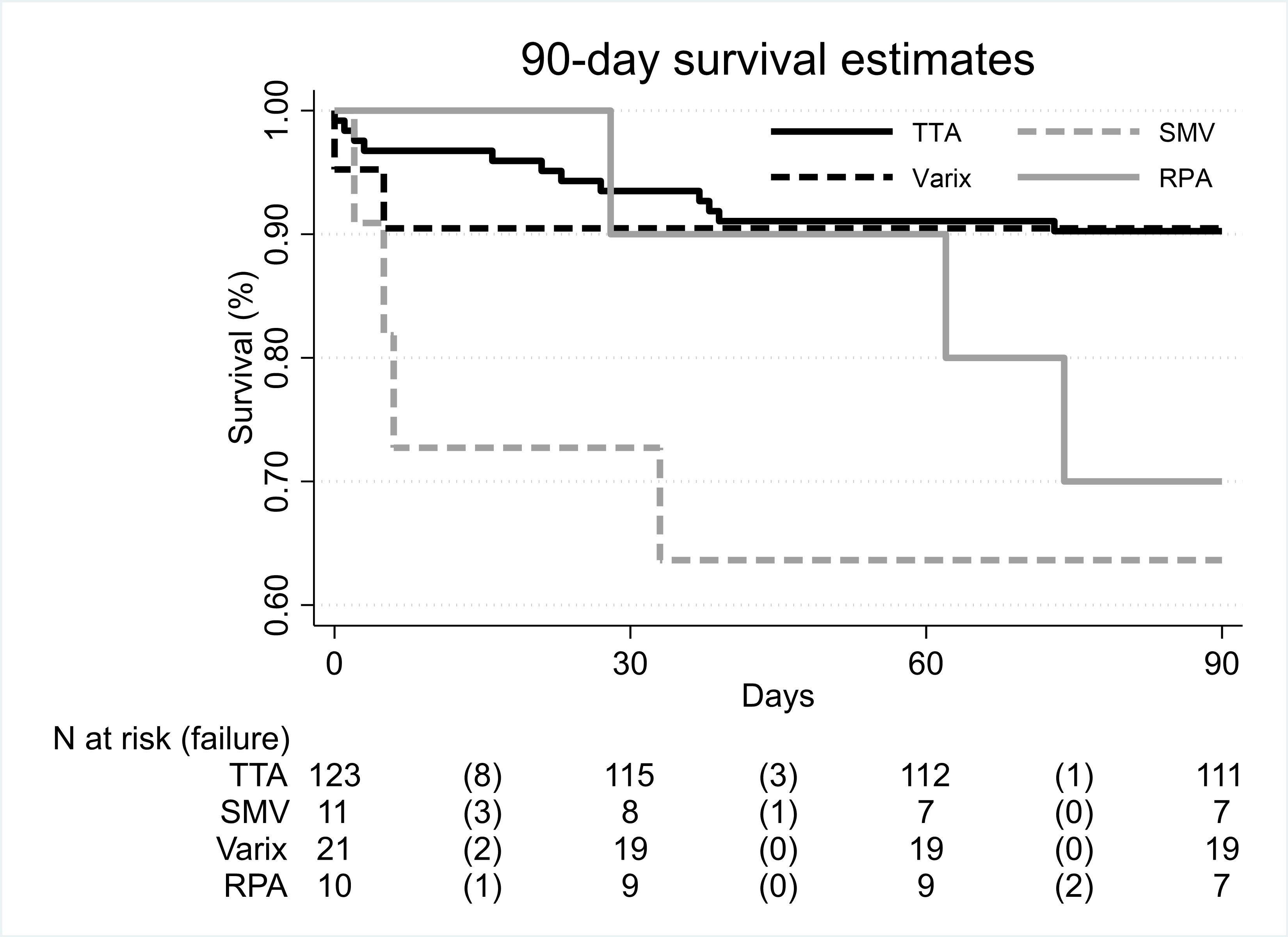

Supplement: Supplementary file 1 [file jcm-12-02457-s001.zip › Supplementary Figure S1.tif]
